# Ethical analysis examining the prioritisation of living donor transplantation in times of healthcare rationing

**DOI:** 10.1136/medethics-2021-107574

**Published:** 2022-01-04

**Authors:** Sanjay Kulkarni, Andrew Flescher, Mahwish Ahmad, George Bayliss, David Bearl, Lynsey Biondi, Earnest Davis, Roshan George, Elisa Gordon, Tania Lyons, Aaron Wightman, Keren Ladin

**Affiliations:** 1 Department of Surgery, Yale University School of Medicine, New Haven, Connecticut, USA; 2 Department of English, Stony Brook University, Stony Brook, New York, USA; 3 Center for Bioethics, Cleveland Clinic Foundation, Cleveland, Ohio, USA; 4 Department of Medicine, Brown Univeristy School of Medicine, Providence, Rhode Island, USA; 5 Department of Pediatrics, Vanderbilt University School of Medicine, Nashville, Tennessee, USA; 6 Department of Surgery, West Virginia University School of Medicine, Morgantown, West Virginia, USA; 7 UNOS Ethics Committee, Richmond, Virginia, USA; 8 Department of Pediatrics, Emory Univeristy School of Medicine, Atlanta, Georgia, USA; 9 Department of Surgery, Northwestern University School of Medicine, Chicago, Illinois, USA; 10 Department of Pediatrics, University of Washington School of Medicine, Seattle, Washington, USA; 11 Department of Community Health, Tufts University, Medford, Massachusetts, USA

**Keywords:** ethics, allocation of health care resources, clinical ethics

## Abstract

The transplant community has faced unprecedented challenges balancing risks of performing living donor transplants during the COVID-19 pandemic with harms of temporarily suspending these procedures. Decisions regarding postponement of living donation stem from its designation as an elective procedure, this despite that the Centers for Medicare and Medicaid Services categorise transplant procedures as tier 3b (high medical urgency—do not postpone). In times of severe resource constraints, health systems may be operating under crisis or contingency standards of care. In this manuscript, the United Network for Organ Sharing Ethics Workgroup explores prioritisation of living donation where health systems operate under contingency standards of care and provide a framework with recommendations to the transplant community on how to approach living donation in these circumstances.

To guide the transplant community in future decisions, this analysis suggests that: (1) living donor transplants represent an important option for individuals with end-stage liver and kidney disease and should not be suspended uniformly under contingency standards, (2) exposure risk to SARS-CoV-2 should be balanced with other risks, such as exposure risks at dialysis centres. Because many of these risks are not quantifiable, donors and recipients should be included in discussions on what constitutes acceptable risk, (3) transplant hospitals should strive to maintain a critical transplant workforce and avoid diverting expertise, which could negatively impact patient preparedness for transplant, (4) transplant hospitals should consider implementing protocols to ensure early detection of SARS-CoV-2 infections and discuss these measures with donors and recipients in a process of shared decision-making.

## Introduction

Living donor kidney and liver transplantation represents the optimal treatment for individuals with end-stage kidney and end-stage liver disease. These life-saving therapies are often contextualised as elective procedures because the donor operation should be conducted in way as to not pressure participation in any manner, yet the transplant procedure in the recipient may have medical urgency based on patient need. The stress on hospital systems due to the COVID-19 2020 Spring surge was unprecedented and legitimate concerns regarding exposure risk, inadequate testing protocols, personal protective equipment and intensive care unit availability required rationing of resources and prioritisation in the types of care delivered.^1–3^ Setting priorities during times of critical resource scarcity is necessary and should be guided by ethical principles that include upholding the ‘rule of rescue’, maximising benefit, safeguarding equitable access for the underserved and promoting intrinsic and instrumental value.^4–6^ With these principles in mind, two key questions remain: What priority should life-saving procedures such as living donor transplants have in a stressed health system? Under what circumstances may it be appropriate to proceed with such procedures?

Immense demands of patients with COVID-19 continue to tax health systems, forcing them to transition to ‘contingency standards of care’ and plan for ‘crisis standards of care’. Crisis standards of care is defined as ‘a substantial change in usual healthcare operations and the level of care it is possible to deliver, which is made necessary by a pervasive (eg, influenza pandemic) or catastrophic (eg, earthquake, hurricane) disaster’.^7^ Within contingency standards of care the spaces, staff and supplies used are not consistent with daily practices but provide care that is *functionally equivalent* to usual patient care.^8^ Health systems sought to preserve functionally equivalent outcomes despite resource limitations by prioritising surgical interventions where medical urgency largely drove immediacy of certain procedures, while those deemed elective, such as living donor transplants, were delayed.

This ethical analysis examines whether proceeding with the living donor transplantation is appropriate when the goal is to provide contingency standards of care—not crisis standards—as long as there are safeguards in place that minimise exposure risk, maintain staff expertise and incorporate engagement of donor/recipient pairs in decisions about their willingness to accept often unquantifiable exposure risks. The aim is not to question prior decisions made under extraordinary circumstances, rather to learn from previous experiences and develop comprehensive decision-making tools to guide actions during future circumstances where healthcare resources are stressed.

## Intrinsic and instrumental value considerations

In determining what priority to assign living donor transplantation, particularly in times of rationing, both intrinsic and instrumental value considerations become relevant. An intrinsic value refers to a normative preference that is inherently appreciated for its own sake, while an instrumental value is prudential, or strategic, and, thus, seeks to achieve a further end. The term *instrumental value* may have different applications in bioethics, but as used in times of rationing healthcare refers to prioritising those—such as living donors—who have the means to save others.^9^ Health has intrinsic value for individuals in society-at-large; it is an ultimate end at which we aim in pursuit of the human good. In contrast, the *manner* in which healthcare ends are procured is typically better understood instrumentally (ie, with an eye towards calculating the strategic costs and benefits of the adopted approach used in decision-making).

Living donor transplantation has obvious intrinsic value, both for the recipient waiting for transplant, and for the living donor who has resolved to donate his, her, or their organ for a recipient’s benefit. At the same time, there are instrumental values to consider in performing living donor transplantation in times of severe resource constraints, where the clear benefits to society of proceeding with a planned transplant must be balanced against critical resources being exigently distributed in other ways. In this case, there are a number of instrumental values to consider. For example, does our allocation of resources appropriately balance the interests of patients of specific disease state, like end-stage organ failure, with the needs of the population-at-large? For example, there were other clinical conditions, for example, cancer patients, who were also subjected to delays in treatment, supply chain disruptions that impacted care and postponement of operative procedures, particularly in situations where radiotherapy or chemotherapy were considered reasonable alternatives.^10^ These delays in cancer treatment were associated with an estimated 20% increase in cancer-attributed mortality.^10^ Thus, the careful balancing of health priorities is needed with considerations of acuity, attributed impact on mortality, options of alternative treatment modalities and the extent of resource allocation for specific interventions. Essentially, this leads to the following questions: Which patient group should be prioritised in times of crisis? What are the potential longer term effects of such prioritisation plans on public perception and trust of the healthcare system? How do such perceptions in the population of potentially prioritising living donation potentially affect living and deceased donation rates?

In considering the prioritisation of living donation in such circumstances, the pragmatism of preserving existing relationships between healthcare providers must also be instrumentally evaluated, for example, between dialysis centres and transplantation centres. There are different levels of scarcity, capacity and expertise that need to be considered and which are likely to be fluid requiring constant re-evaluation. For example, a living donor recipient who is predicted to require an ICU bed may or may not be an appropriate candidate dependent on several health system factors that could change in a short amount of time.

Ideally, we would pursue a policy that reconciles intrinsic values with our understanding of the greater good, and where instrumental considerations align and reinforce intrinsic goals. In considering how intrinsic values cohere with instrumental values, during times of crisis, it may be useful to identify critical thresholds beyond which we revisit standing policies in order to make sure that all of the relevant considerations are being taken into account. These types of value assessments require a determination of a given need and the social context of a donation. These can only be fully understood through a framework that supports an open dialogue with patients, their respective donors and considerations of the potential impact to non-transplant patients whose care may be affected.

## An ethical framework balancing risk with donor and recipient autonomy

**Figure 1:**
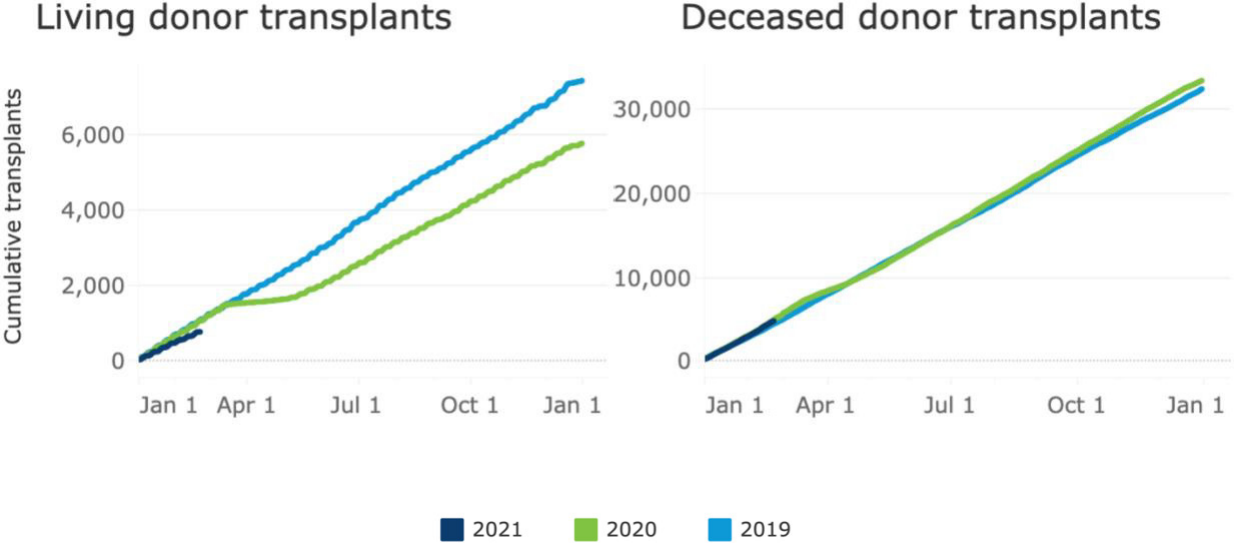
Number of living donor and deceased donor transplants in 2019, 2020 and 2021 (to date). Adopted from unos.org/covid/ (accessed April 30, 2021).

The rationing of healthcare in times of resource scarcity is both required and ethical.^11^ During the Spring 2020 surge, 96.7% of US Hospitals suspended elective procedures.^12^ As figure 1 shows, many of these hospitals designated living donor transplants as elective, despite the fact that transplant procedures are classified as tier 3b (high medical urgency—do not postpone) by Centres for Medicare & Medicaid Services (CMS).^13^ Currently, there is increasing discussion on how rationing decisions were made, the impact of postponing procedures on patients and if continuing certain elective procedures in times of health rationing is appropriate.^14^ In this context, it is important to develop an ethical framework that supports proceeding with procedures such as living donor transplants, under conditions of uncertainty and when contingency standards of care are in effect. It is important to recognise that decisions made to delay living donor transplants were conducted under extreme and unprecedented circumstances, where the trajectory of the surge was uncertain and the capacities of health systems rightly prioritised care delivery to support the needs of the population-at-large.

The ethical principles that govern organ transplantation should ideally balance considerations of utility, justice (equity) and respect for persons (autonomy).^15^ While the tension between utility and justice is arguably heightened in times of severe health resource scarcity, autonomy considerations should have greater emphasis when the risks are not quantifiable, yet the benefits are well established. Indeed, engaging patients in shared decision-making is appropriate when risks are uncertain and evidence-based medical outcomes are lacking.^16–19^ During the Spring 2020 suspension of living donor transplantation due to the COVID-19 surge, transplant professionals pointed to safety considerations for both the donor and recipient as a primary driver for their decision to delay transplantation.^20^ For donors in particular, safety considerations are of paramount concern for transplant teams. However, as the exposure and outcome risks are ambiguous and dependent on several factors, including the local density of infections, engaging donor/recipient pairs in discussions regarding their perspectives of accepting risk promotes autonomy, enhances patient-centred care and should have greater emphasis on clinical care decisions.^21 22^ In the context of comprehensive informed consent including shared decision-making and under contingency standards of care, there may be potential room for discretionary continuation of living donor transplantation under specific conditions in order to best serve the interests of liver and kidney patients, the transplant community and to promote donor/recipient autonomy.

## Temporary suspension of living kidney donation during COVID-19

In Spring 2020, 81% of transplant centres in regions of the country with a high cumulative COVID-19 prevalence (≥500 cases/100k population) chose to internally suspend their living donor kidney programmes, although none formally inactivated their programmes with Organ Procurement and Transplantation Network/United Network for Organ Sharing.^20^ Transplant programmes reported concerns regarding donor (85%) and recipient (75%) safety, as well as elective case restrictions (47%), as primary reasons for suspending living donor kidney cases.^20^

Boyarsky *et al* have analysed data from the Scientific Registry of Transplant Recipients on the early effect of COVID-19 on kidney transplant outcomes. They show that new listings, deceased donor transplants and living donor transplants declined by 18%, 24% and 87%, respectively (15 March 2020 to 30 April 2020). States with the highest rates of COVID-19 cases had the lowest rates of living kidney donor transplants than expected (IRR=_0.00_0.01_0.05_), while these same states had a 2.2-fold higher observed waitlist mortality (IRR=_1.88_2.22_2.62_).^23^

The first surge was associated with a decrease in total living donor kidney transplants performed compared with 2019, while deceased donor volume was largely unaffected (figure 1). This could represent the fact that the Spring 2020 surge was geographically limited, resulting in the suspension of living donor transplant programmes locally, while deceased donor grafts continued to be accepted in less affected regions. This is supported by the observation that deceased donor recoveries across organ types were reduced and disproportionately impacted in the US Northeast during the Spring 2020 surge.^24^ Currently, no analysis provides a causal association between suspension of living donor kidney programmes and the observed rise in waitlist mortality. Additionally, it is also unknown how many approved donor/recipient pairs, whether scheduled for surgery or not, ultimately did not undergo living donor transplantation due to restrictions imposed by the COVID-19 surge and the impact of this on waitlist mortality.

It is now evident that both in the USA and internationally, risks of proceeding with living donor transplant with its potential impact on patients and healthcare resources need to be balanced with the risks of continued dialysis where challenges to social distancing, increased demand and worse outcomes related to COVID-19 exposure are increasingly understood.^18 25 26^ Martin *et al* also make the point that cessation of living donor transplant is particularly relevant in areas where dialysis is not readily available or constrained due to local resources.^18^ Thus, there are several factors, many of them dependent on changing local conditions, that need to be considered when balancing exposure risks with proceeding with living donor transplantation in a safe manner.

## Living donor liver programmes and the ‘rule of rescue’

Although there was no appreciable difference between 2019 and 2020 in the number of living donor liver cases performed, subsequent surges of the ongoing COVID-19 pandemic may pose a threat to the volume of living liver donor transplants given local conditions. From 15 March 2020 to 31 August 2020, Strauss *et al* show that liver transplants from living and deceased donors were 49%, and 9% lower compared with historical trends, respectively.^27^ These observed reductions largely recovered to expected norms by August of 2020.^27^ Unlike patients with end-stage kidney disease who have the alternative of dialysis, patients with end-stage liver disease do not have a similar life-sustaining therapy to manage them in the face of critical delays brought about by COVID-19 restrictions. However, greater resource requirements, primarily from blood and ICU utilisation, are critical considerations in determining whether liver transplantation, and in particular those from living donors, should continue under contingency standards of care.

We posit that there are indeed harms to delaying living liver donor transplants because this subset of patients does not have options for sustaining survival other than transplantation and, as such, represent a patient population with a high level of medical urgency. From an ethical perspective, the rule of rescue supports continuance of these procedures as it suggests that it is an imperative to advocate on behalf of an individual patients’ need who face a clear fatal outcome, regardless of expense.^28^ The key considerations include the predicted mortality of patients without liver transplant and a transplant hospitals’ capacities, specifically blood product and ICU availability. In this context, it is important to recognise that many potential recipients, particularly those from living donors, may be sicker than a Model for End-Stage Liver Disease (MELD) score may predict and, therefore, the urgency of proceeding with transplantation in times of healthcare scarcities may be ethically justifiable. However, it is also recognised that there is a subset of liver transplant recipients who may be stable at a lower MELD score or have a transplant indication, such as hepatocellular carcinoma, where a delay in a living donor transplant may be reasonable.

## Recommendations for transplant community

In light of the ethical analysis above, we propose some modest conclusions intended to be used as resources of guidance. These recommendations apply to transplant hospitals experiencing contingency standards of care and may not be applicable in conditions guided by crisis standard of care.

1. Living donor transplants represent an important option for individuals with end-stage liver and kidney disease and should not be suspended uniformly when the aim is to provide contingency standards of care. Decisions to delay living donation may be appropriate given local conditions. However, these decisions should have on-going evaluation, be communicated transparently and include timely dialogue with patients.
2. Exposure risk to SARS-CoV-2 should be balanced with other risks, such as exposure risks at dialysis centres. Because many of these risks are not quantifiable and variable given local case rates, donors and recipients should be engaged in the process of documented shared decision-making, in order to understand their perspectives of what constitutes risk as well as to come to a mutual appreciation of the social context of donation which informs the willingness to undertake these risks.
3. Transplant hospitals should strive to maintain a critical transplant workforce in order to avoid diverting this unique expertise in a manner, which could negatively impact patient preparedness for transplant. Under crisis standards, it is understandable that every available resource is optimised to provide prioritised care. However, under contingency standards, transplant centres continue to provide equivalent services and, as such, should do so with an optimised workforce familiar with the complexities of waitlist management, transplant organ offers and post-transplant management.
4. As the understanding of COVID-19 outcomes in immunosuppressed patients continues to evolve,^29^ transplant hospitals should consider implementing protocols to ensure early detection of SARS-CoV-2 infections and include these measures as a discussion with patients during a process of shared decision-making.

## Conclusion

Many transplant hospitals designate living donor transplantation as an elective procedure, which makes these operations susceptible to temporary suspensions in favour of procedures categorised as emergent or urgent. Although CMS designates transplantation at a high level of medical urgency (tier 3b), the elective nature of living donor transplantation reflects the general agreement that a living donor undergo’s surgery mainly to benefit others and should so without pressure or urgency. It is understandable that under crisis standards, living donor transplant procedures should be delayed in favour of allocating crucial resources to the population-at-large. Under contingency standards, which offers functionally equivalent care delivery, additional considerations applicable to a specific environment are necessary. For example, to assure standards of contingency during an outbreak of COVID-19 hospitalisations, additional protocols to minimise infectious transmission and to assure an adequate transplant workforce are key elements that need to be discussed with patients. Given the uncertainty surrounding the risks and outcomes associated with hospital exposure to SARS-CoV-2, advocating for continuance of these procedures under contingency standards of care should be undertaken by engaging donor/recipient pairs in a process of shared decision-making and within a framework of comprehensive informed consent. Although this proposed ethical framework was developed in the context of the COVID-19 pandemic, it may also be applicable in situations where local healthcare resources are strained, where continency standards are in effect, and where critical decisions regarding healthcare priorities need to be made.

## Data Availability

All data relevant to the study are included in the article.
